# A Non-Stationary Relationship between Global Climate Phenomena and Human Plague Incidence in Madagascar

**DOI:** 10.1371/journal.pntd.0003155

**Published:** 2014-10-09

**Authors:** Katharina S. Kreppel, Cyril Caminade, Sandra Telfer, Minoarison Rajerison, Lila Rahalison, Andy Morse, Matthew Baylis

**Affiliations:** 1 LUCINDA group, Institute of Infection and Global Health, Department of Epidemiology and Population Health, University of Liverpool, Neston, United Kingdom; 2 Department of Geography and Planning, School of Environmental Sciences, University of Liverpool, Liverpool, Merseyside, United Kingdom; 3 Institute of Biological and Environmental Sciences, University of Aberdeen, Aberdeen, United Kingdom; 4 Unité Peste - Institut Pasteur de Madagascar, Antananarivo, Madagascar; 5 Centers for Disease Control and Prevention, Division of Bacterial Diseases, Atlanta, Georgia, United States of America; 6 Health Protection Research Unit in Emerging and Zoonotic Infections, University of Liverpool, Neston, United Kingdom; Yale University, United States of America

## Abstract

**Background:**

Plague, a zoonosis caused by *Yersinia pestis*, is found in Asia and the Americas, but predominantly in Africa, with the island of Madagascar reporting almost one third of human cases worldwide. Plague's occurrence is affected by local climate factors which in turn are influenced by large-scale climate phenomena such as the El Niño Southern Oscillation (ENSO). The effects of ENSO on regional climate are often enhanced or reduced by a second large-scale climate phenomenon, the Indian Ocean Dipole (IOD). It is known that ENSO and the IOD interact as drivers of disease. Yet the impacts of these phenomena in driving plague dynamics via their effect on regional climate, and specifically contributing to the foci of transmission on Madagascar, are unknown. Here we present the first analysis of the effects of ENSO and IOD on plague in Madagascar.

**Methodology/principal findings:**

We use a forty-eight year monthly time-series of reported human plague cases from 1960 to 2008. Using wavelet analysis, we show that over the last fifty years there have been complex non-stationary associations between ENSO/IOD and the dynamics of plague in Madagascar. We demonstrate that ENSO and IOD influence temperature in Madagascar and that temperature and plague cycles are associated. The effects on plague appear to be mediated more by temperature, but precipitation also undoubtedly influences plague in Madagascar. Our results confirm a relationship between plague anomalies and an increase in the intensity of ENSO events and precipitation.

**Conclusions/significance:**

This work widens the understanding of how climate factors acting over different temporal scales can combine to drive local disease dynamics. Given the association of increasing ENSO strength and plague anomalies in Madagascar it may in future be possible to forecast plague outbreaks in Madagascar. The study gives insight into the complex and changing relationship between climate factors and plague in Madagascar.

## Author Summary

Plague is a vector-borne bacterial infection with rodents and their fleas as its principal hosts. Transmission to humans occurs via the bite of an infected flea. In the highlands of Madagascar, plague is endemic and more than one hundred human cases are reported every year. Global climate is known to affect many infectious diseases and has been shown to affect plague incidence in other areas of the world. The ENSO and the IOD are global climate drivers affecting rainfall and temperature in Madagascar. Our study investigates the effect of global climate drivers on human plague incidence on the island. We found a link between ENSO, IOD, temperature and precipitation and plague incidence throughout the 48-year time-series although it was not constant over time. The correlation between ENSO and plague turned from weakly positive to strongly negative and then to positive, and the association with the IOD became stronger with time. We demonstrate that during periods of high ENSO intensity, plague incidence is likely to increase via ENSO's impact on temperature and precipitation. This shows that climate indices can be a tool to help predict human plague incidence.

## Introduction

Plague is a vector-borne, highly virulent zoonotic disease present today in the Americas, Asia and Africa. It is caused by infection with the bacterium *Yersinia pestis*, which triggers serious illness with up to seventy percent case fatality in human populations if left untreated. The infection is easily treated with antibiotics, yet these are often difficult to access in time in low income settings.

The increasing frequency of the disease in many parts of the world [1], [2], [3] has been partly attributed to changes in climate [4]. Presently Africa accounts for more than ninety percent of all human plague cases reported worldwide. Within African countries, the majority of cases are reported from Madagascar and the Democratic Republic of Congo [5], with Madagascar reporting almost one third of human cases worldwide. Plague is endemic in the highland region of Madagascar and more than one hundred human cases are reported every year, though the true number of cases is likely to be higher. The reasons for such pronounced foci in these areas include extreme poverty and lack of health infrastructure, as well as unique climate features.

*Yersinia pestis* bacteria are transmitted between rodent hosts via their fleas; humans are accidentally infected when in contact with rodent fleas or infected animal tissue. Like many vector-borne diseases, plague's occurrence varies temporally and spatially on a variety of scales. The primary mechanisms directing this heterogeneity are thought to be driven by local variation in factors such as temperature and rainfall. For example temperature influences the rate of development of the bacterium in the flea, and the survival and development times of the fleas themselves, while precipitation affects food availability and thus fecundity of the rodent host [6], as well as having impacts on habitat characteristics and human-rodent contact (via rodent responses to flooding).

Seasonal climate in many parts of the world is affected by the El Niño Southern Oscillation (ENSO), a periodic fluctuation in sea surface temperature and air pressure in the Pacific Ocean which modifies the general flow of the atmosphere in the tropics. Warm (cold) phases of the ENSO, called El Niño (La Niña), are associated with a warming (cooling) of the tropical troposphere [7]. ENSO impacts on the climate of Madagascar in a manner similar to that observed over southern Africa by Nicholson and Selato [8]. In general, an austral spring-summer El Niño starting from September causes warmer and drier conditions than usual during the austral summer-autumn four to seven months later, and cooler and wetter conditions eight to twelve months later. La Niña has the opposite effect, leading to wetter and cooler, followed by drier and warmer, conditions than average.

The Indian Ocean Dipole (IOD) [9] is a periodic fluctuation in the relative sea surface temperatures of the western and eastern parts of the Indian Ocean. As the western pole of the IOD is located near to Madagascar, IOD events affect the convection locally and in turn influence the climate of the island. Thus, a positive IOD event is associated with warmer and wetter conditions over the island, while the opposite is true for a negative event [9].

We present here the first analysis of the effects of ENSO and IOD on the temporal distribution of confirmed human plague cases in Madagascar. We use a forty-eight year time-series from 1960–2008. First we identified periods of anomalous incidence and evaluated their occurrence against any coinciding trends of various climate variables. Second we use wavelets to investigate the strength and direction of association between the incidence of plague and ENSO, IOD, temperature and precipitation. Wavelet analysis allows detection of relationships between two time series in time-frequency space. Our approach uses analytical methods which allow a more thorough analysis than the usual identification of long-term unidirectional trends.

## Methods

### Plague incidence data

Data on all confirmed human plague cases reported in Madagascar from 1956 to 2008 were made available by the World Health Organisation Plague Reference Laboratory of the Institut Pasteur de Madagascar. A time-series of monthly incidence from 1960 to 2008 was created using the date of onset of symptoms for each confirmed bubonic, pneumonic or septicaemic case, 5-yearly human population growth estimates from the United Nations and the last population census from 1993 [10]. Incidence was calculated for each month using the number of cases, multiplying it by 100,000 for scale and dividing it by the relevant time-specific population estimate. Data from before 1960 was available but was omitted due to vaccination campaigns which ceased in 1959. For effective immunisation, people have to be re-vaccinated yearly. Malaria prevention programs, which occur in some areas of Madagascar and use indoor residual spraying of insecticide, have the potential to impact flea populations. However, this could not be quantified or corrected for.

#### Plague seasonality and incidence anomalies

The seasonality of human plague incidence was obtained by taking the average monthly values over all years to get monthly means. Monthly plague incidence anomalies were obtained for the entire time series by applying a four year moving average for each month to the human incidence data and subtracting it from the original incidence value to establish the deviation of the data point from the mean value.

### Explanatory variable datasets

Re-calculated climate variables were downloaded via the climate explorer website [11] based on the National Center for Environmental Prediction (NCEP) and the National Center for Atmospheric Research (NCAR) reanalysis data [12] or the Centre of Environmental Data Archival website (precipitation) [13]. For the El Niño Southern Oscillation variable the index of the Japan Meteorological Agency (JMA) was retrieved from the Centre for Ocean-Atmospheric Prediction Studies website [14]. Monthly surface temperature and precipitation anomalies for the geographical area of Madagascar (42.18°E–49.68°E; 24.76°S-11.42°S) for the period 1960–2008 were used to describe seasonal cycles and in wavelet analyses.

#### Temperature and precipitation anomalies and seasonality

The seasonal cycle of temperature and precipitation variables was obtained by averaging monthly values over the entire study period based on the CRUTS3.1 dataset [13].

Temperature and precipitation anomalies were produced by calculating the mean for each calendar month across the whole time-series and subtracting it from the mean value for each particular month based on the NCEP-NCAR reanalysis [12].

#### El Niño Southern Oscillation

The ENSO phenomenon is represented here as spatially averaged monthly sea surface temperature (SST) anomalies over the tropical Pacific (4°N to 4°S and 150°W to 90°W) with an applied 5-month running mean to smooth out possible intra-seasonal variations for the period 1960–2010.

#### The Indian Ocean Dipole

The IOD is represented here by the Dipole Mode Index (DMI) which is the difference between the Western Tropical Indian Ocean (50°E–70°E and 10°S-10°N) sea-surface temperature index and the South-eastern Tropical Indian Ocean (90°E–110°E and 10°S-0°N) sea-surface temperature index. Here too, a 5-month running mean was applied.

#### Standardisation of anomaly time-series

Anomaly time-series of all explanatory variables and plague incidence anomalies were standardised by dividing each monthly value by the maximum of the absolute value across the whole time period to allow for graphical comparison.

### Plague anomaly analysis

A plague incidence anomaly of >0.1 or <−0.1 was used to define months of anomalously high/low plague incidence respectively. When months of anomalously high or low plague incidence were sequential or less than 3 months apart, they were considered to be part of the same plague event; otherwise, they were treated as separate plague events. A plague event could therefore be for a single month or for several months. For plague events of >1 month duration, the single month which had the maximum or minimum incidence anomaly (depending on whether it was a high or low incidence event) was identified.

We tested for three types of association between plague incidence anomaly and the explanatory variables: (i) using Analysis of Variance, to test for associations between positive or negative plague anomalies and the value of the explanatory variables (JMA, IOD, temperature, precipitation) in the month or peak month of a plague event; (ii) using Analysis of Variance, to test for associations between positive or negative plague anomalies and the mean value of the explanatory variable for the year centred on the month or peak month of a plague event; (iii) using Fisher Exact Test, to test for associations between positive or negative plague anomalies and positive or negative trends in the explanatory variables for the year centred on the month or peak month of a plague event. For the third analysis, we obtained the sign of the linear regression coefficient for the trend of the 12 monthly values (with the 7^th^ month as the peak plague anomaly month) and use this to determine if the explanatory variable was tending to increase (positive coefficient), or decrease (negative coefficient), at the time of a plague event.

### Wavelet analysis

Time-series analysis using wavelets was undertaken on ENSO/IOD/temperature/precipitation and human plague incidence anomaly datasets spanning the 48-year study period. The objective was to establish any associations in time between plague and the climate variables, and, if present, their direction and periodicity. Wavelet analysis [15], [16] is a powerful means to identify statistical relationships between signals, and is especially useful when there is non-stationarity; i.e. the periodicity changes with time [15], [17]. Wavelet analysis is a widely recognised tool to investigate temporal dynamics of infectious diseases [18], [19], [20], [21], [22], but has never been used to study the relationship between climate variables and plague in Madagascar.

To detect temporal patterns, their variations and coherence, we applied wavelet analysis according to the methods of Grinsted [17] using the software R v. 2.15 [23] and Matlab 8.0 v. R2012a with Wavelet Toolbox [24].

The following procedure was used. First, the five variables of interest - precipitation, temperature, ENSO, IOD and plague incidence - were tested for normality, and where necessary, normalised using a Johnson transformation [25]. A low-pass Gaussian filter was used to remove the intra-seasonal variability in the time-series. Second, the stationarity or non-stationarity of each variable was determined using continuous wavelet decomposition. Each time-series was decomposed and the continuous wavelet transform plot was examined to confirm the presence of high significant variance and to establish its periodicity and any changes within the time-series.

Third, the strength of any relationship between certain pairs of variables was investigated using cross-wavelet analysis, which identifies high common power between two signals (time frames where both signals vary together). The direction of the vectors reveals information about the phase relationship (i.e. time-lags) between two time-series. A vector pointing to the right indicates the time-series cycle in-phase and a vector to the left indicates cycling in anti-phase. Thus, any red/yellow areas within figures show periods during which the signals cycle with high common power with the vectors signifying the phase and the time lag.

Lastly, the presence and direction of any relationship (positive/negative) between variables was established using wavelet coherency analysis, with vectors again indicating the direction of association and time lag. Here, a vector pointing to the right indicates positive association and a vector to the left indicates negative association. Downward or upward pointing vectors reveal information about which time-series leads. Wavelet coherence can be understood as an association between signals in the power spectrum space. It identifies regions of the power-space spectrum where vectors point in one direction.

For both cross-wavelet and wavelet coherence analysis, information on time-lags between the time-series is revealed by the direction of the vectors. If two signals cycle with significant common power in cross-wavelet analysis or show association in wavelet coherence analysis with a 2 year periodicity, a right-pointing vector means they are cycling in phase; a left pointing vector means they are cycling in anti-phase (one lags the other by half the periodicity, i.e. 1 year); a downward pointing vector means the first signal leads the second signal by one quarter of the periodicity (half the difference between in-phase and anti-phase, i.e. 6 months; and so forth) while an upward pointing vector means the second signal leads the first signal by one quarter periodicity.

A cone of influence (COI) was applied to the cross-wavelet and wavelet coherence transforms to mark the limits of the time scale within which signal behaviour can be discussed with confidence. The statistical significance level of the wavelet coherence is estimated using Monte Carlo methods [16]. Further information about these methods is provided by Grinsted et al. [17].

## Results

### Plague incidence and seasonality

**Figure 1:**
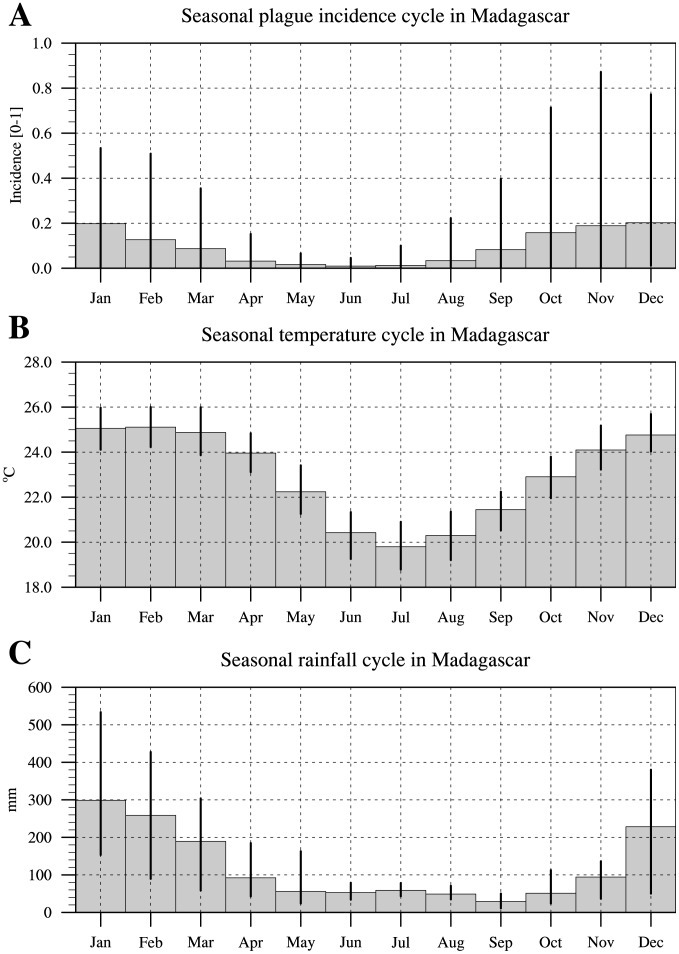
Seasonality of human plague incidence, temperature and rainfall. (A) Seasonal plague incidence cycle in Madagascar for the period 1960–2008 (B) Seasonal temperature cycle over Madagascar for the period 1960–2008 (bars) based on the CRUTS3.1 dataset [24]. (C) Seasonal rainfall cycle over Madagascar for the period 1960–2008 (bars) based on the CRUTS3.1 dataset. The vertical lines depict the minimum/maximum observed over the period for a given month.

Plague cases occur year-round but there is strong seasonality, with most cases occurring from September to March (the austral summer) and reaching a peak from November to January (Figure 1A). The largest inter-annual variability in plague cases occurs from October to December. The seasonal cycles of temperature and rainfall show highest values from November to April, and December to March respectively (Figure 1B and 1C). December to March are also the months with the largest inter-annual range in rainfall. The inter-annual range in temperature values is approximately equal in all months. Thus, the warm, wet season is the time of the highest plague incidence and greatest variation in incidence.

#### Anomaly time-series

**Figure 2:**
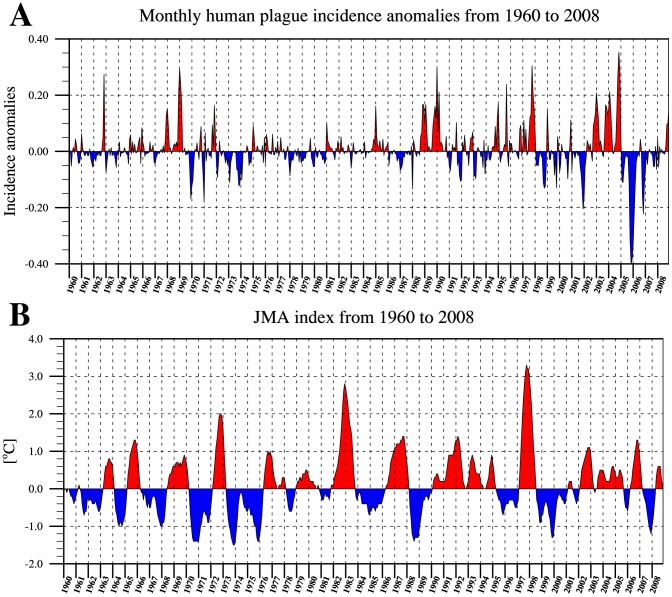
Monthly anomaly time-series of human plague incidence and JMA (ENSO index). (A) Monthly plague incidence anomalies in Madagascar for the period 1960–2008 (B) Monthly JMA index for the period 1960–2008. Positive values are depicted in red, negative values are blue.

Annual anomalies in plague incidence were mostly small (<0.1) but in some years were as high as 0.3 or as low as −0.4 (Figure 2A). Both the frequency and magnitude of anomalies appears to have increased in the last two decades. Notably, despite filtering the data, there remains a trend towards higher variance later in the time-series. An increase in the number of confirmed plague cases can also be seen towards the end of the incidence time-series (Figure S1).

In the ENSO anomaly time-series (JMA) the usual annual range of positive and negative events reaches from just above 1.0 in El Niño years to −1.0 in La Niña years respectively. At the beginning of the time-series from 1960 to 1975 La Niña events dominate with only one strong El Niño event in 1972. Thereafter in the time period 1976 to 1981, no strong anomalies occur until in 1982/83 the second strongest positive anomaly of the time-series occurs, with a value of ∼2.8. The early 1990s are dominated by moderate positive anomalies until after a weak negative period in 1995 and 1996 the strongest El Niño on record can be seen in 1997/98 reaching a value of 3.2 (Figure 2B).

The IOD index (DMI) anomaly series shows an unusually high positive range in 1961/62 followed by alternating positive and negative events of similar ranges (≥2.2; ≤−1.4). A positive event in 1994/95, reaching a value of 2.8 precedes a negative anomaly value of −2.8 in the following year. During the period of the strongest El Niño event in the time-series (1997/98), the IOD also shows its largest positive range with an anomaly value of 3.2 (Figure S2).

The anomalies for temperature show negative ranges until the late 1970s and early 1980s after which the time-series is dominated by positive values (Figure S3). In other words, there has been a gradual increase of Madagascar's temperatures.

Precipitation shows regularly alternating negative and positive ranges in fast succession. Positive anomalies are more frequent than negative anomalies from mid-1978 until 1985, and again from 1993 until the end of the time-series (Figure S4).

### Graphical comparison of time-series

#### Comparison of anomaly time series

**Figure 3:**
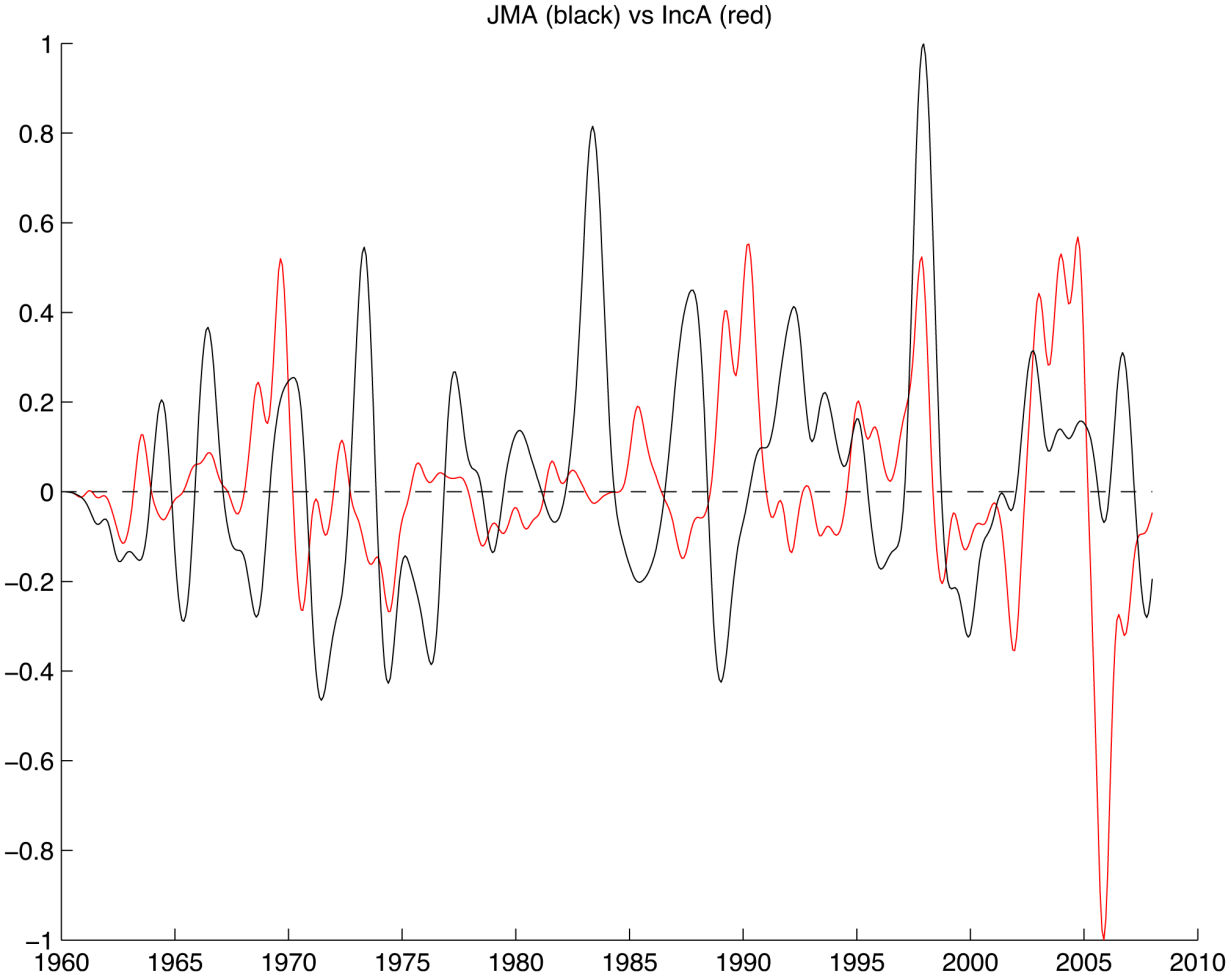
1D plot of the monthly JMA index (black) and the monthly filtered plague incidence anomalies (red) for the period 1960–2008.

From the 1960s to the mid-1970s, plague incidence anomalies precede ENSO anomalies by about a quarter period (Figure 3). For the 1980s the indices appear to be in anti-phase. However, from the mid-1990s until almost the end of the time series ENSO and plague incidence seem to be in phase. The IOD and plague incidence anomaly time-series show no evident pattern until the period from the mid-1990s to 2007 when they vary in-phase (Figure S5).

Temperature anomalies and plague incidence anomalies appear to vary anti-phase from the 1960s to early 1990 (Figure S6). In later years temperature seems to lag incidence by about 6 months. Precipitation anomalies and plague incidence anomalies do not show any clear pattern except in the early 2000s when they seem to vary in-phase (Figure S7).

No clear patterns are apparent between ENSO and precipitation or IOD and precipitation by graphical comparison of the anomaly time-series (Figure S8 and Figure S9). However, ENSO seems to lead temperature anomalies by about a year throughout the time-series (Figure S8). For IOD and temperature there is an in-phase relationship, with IOD leading by about 6 months until the early 1990s when the signals start to vary in anti-phase ().

#### Association between positive/negative plague incidence anomalies and explanatory variables

We tested this association as follows. Table 1 lists 29 periods of anomalously low and high incidence identified from Figure 2A. Of 12 negative plague anomalies (anomaly <−0.1), 9 are associated with decreasing JMA and only 3 are associated with increasing JMA. By contrast, of 17 positive plague anomalies (anomaly >0.1), only 7 are associated with decreasing JMA and 11 are associated with increasing JMA. In other words, negative plague anomalies tend to occur during periods of decreasing JMA (strengthening La Niña), and positive plague anomalies tend to occur during periods of increasing JMA (strengthening El Niño). This association is significant at the 90% level (Fisher Exact Test, P = 0.07). We detect no equivalent associations between plague incidence anomaly and the trend in the anomalies of IOD, precipitation or temperature.

**Table 1:** Periods of anomalously low and high incidence and the associated trend in ENSO (JMA) for the period 1960–2008.

Period of anomaly		Month of greatest anomaly	Incidence anomaly	Sign of JMA trend
Nov	1962	Nov	0.2741	+
Jan	1969	Jan	0.2942	+
Dec	1969	Dec	−0.1686	−
Jan	1971	Jan	−0.1806	+
Nov	1971	Nov	0.1633	+
Feb	1973	Feb	−0.1111	−
Nov	1973	Nov	−0.1230	−
Jan	1981	Jan	0.1029	−
Jan	1985	Jan	0.1609	−
Jan	1988	Jan	−0.1335	−
Feb	1989	Feb	0.1676	+
Jan	1990	Jan	0.3022	+
Aug	1991	Aug	0.1026	+
Dec	1991	Dec	−0.1065	+
Jan	1995	Jan	0.1716	−
Sep	1995	Sep	0.2383	−
Feb	1997	Feb	0.1109	+
Oct	1997	Oct	0.3042	+
Oct	1998	Oct	−0.1306	−
Jan	1999	Jan	0.1487	−
Oct	1999	Oct	−0.1297	−
Dec	2000	Dec	0.1115	+
Dec	2001	Dec	−0.2043	+
Jan	2003	Jan	0.2084	−
Feb	2004	Feb	0.2130	−
Nov	2004	Nov	0.3534	+
Mar	2005	Mar	−0.1115	−
Nov	2005	Nov	−0.4003	−
Nov	2006	Nov	−0.2217	−

One-Way ANOVA was used to test for associations between positive or negative plague anomalies and the value of JMA, IOD, temperature and precipitation in the anomaly month, and the average of these variables in the year centred on the anomaly month. No associations were detected for JMA, IOD or temperature; however, positive plague anomalies were significantly associated with higher average rainfall, and negative plague anomalies with lower average rainfall (positive/negative plague anomalies, mean rainfall anomaly of year = 0.137/−0.120; F_1,28_ = 6.01, *P* = 0.021).

These results suggest that positive plague anomalies (times of higher than usual plague incidence) are in association with increasing JMA index and higher than usual rainfall.

### Wavelet analyses

#### Continuous wavelet transform of plague incidence anomalies

**Figure 4:**
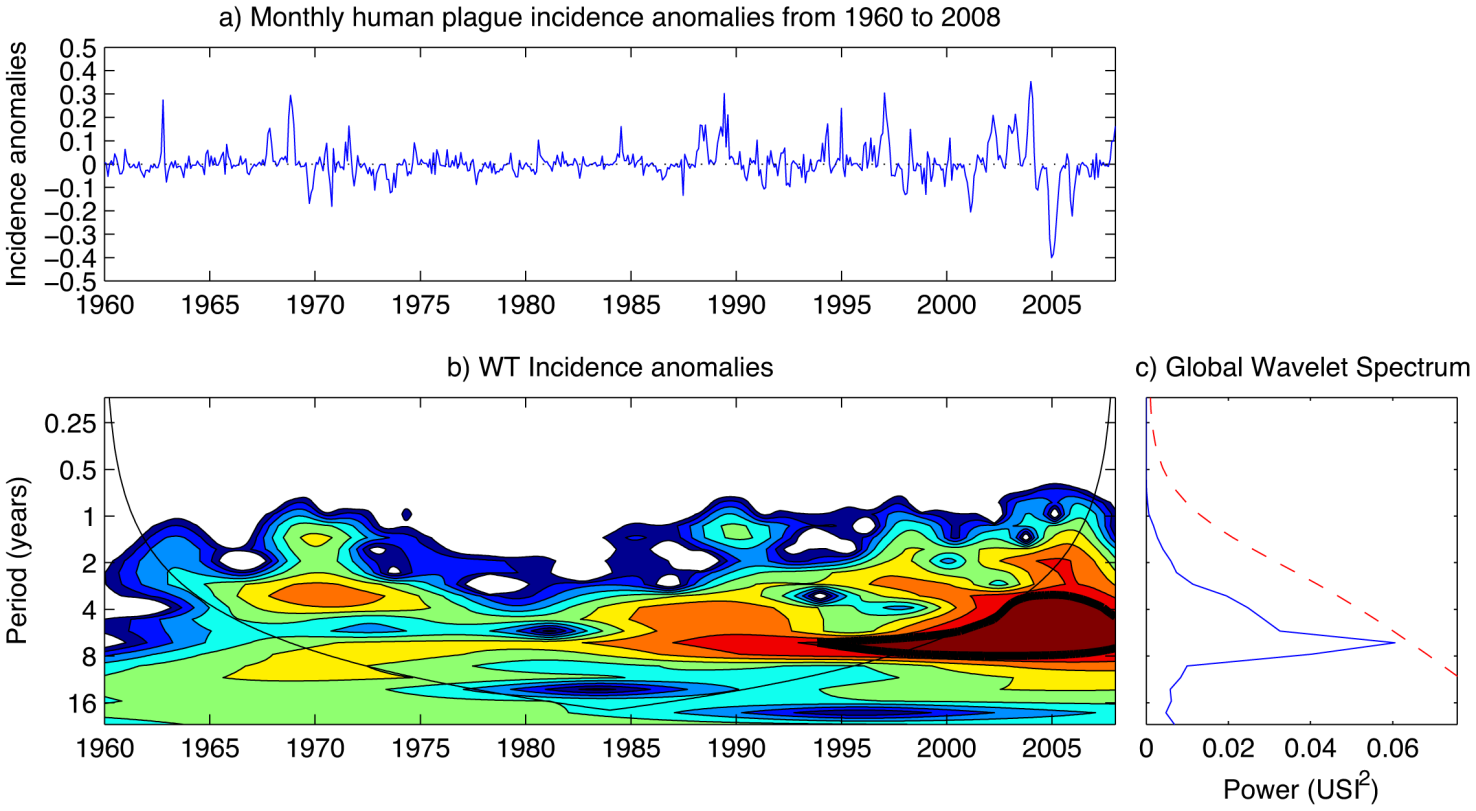
Plague incidence anomalies data. (A) Monthly human plague incidence anomalies from 1960 to 2008 for Madagascar. (B) Associated continuous wavelet power spectrum. The dark contours denote power significance at the 95% level. (C) Global wavelet spectrum. A Gaussian filter has been applied before calculating the continuous wavelet power spectrum and the global spectrum.

The continuous wavelet power spectrum is shown in Figure 4B. There is good correspondence between large variance in the continuous wavelet power spectrum and the incidence anomalies time-series in Figure 4A. Figure 4B shows non-stationarity of the incidence series and confirms the presence of some significant variance around 1970 and highly significant variance from 1985 onwards, as indicated by the yellow-red areas. This variance changes periodicity from 2 to 4 years in around 1970 to 1.5 to 8 years. Figure 4C shows the integrated magnitude of the periodicities identified in Figure 4B with a peak centred around 6–7 years.

#### Continuous wavelet transforms of explanatory variables

The wavelet power spectrum for the ENSO index (JMA) depicts high periodic variance in the 2–5 year band from 1960 onwards, indicating significant 2–5 year periodicity in sea surface temperatures. This variation slowly shifts to a 2–7 year periodicity by the end of the time-series (Figure S10).

The wavelet power spectrum for the IOD index (DMI) shows significant periodic variance in the 1.5 to 6 year band with increased significance from 1990 onwards (Figure S11). The wavelet power spectrum for the temperature anomaly data set shows a band of significant variance throughout the time-series with a periodicity of 8–12 years starting in the early 1970s. Additionally, significant periods of variance are present during the first 30 years from 1960 to 1990. Here the periodicity shifts from a range of 1 to 7 years until 1972 to a slightly smaller range of 3 to 5 years until 2000 (Figure S12).

The wavelet power spectrum for the precipitation anomaly data set shows significant, yet weak variance in the 1–2 year band, denoting inter-annual variability throughout much of the time-series, but no significant times of variance at higher periods (Figure S13).

#### Influence of ENSO on plague incidence anomalies

**Figure 5:**
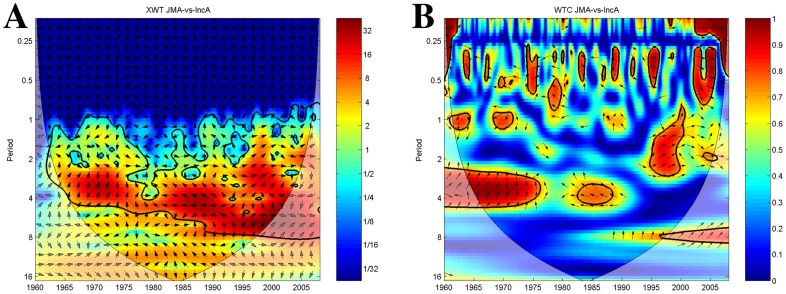
Plague incidence anomalies and ENSO index time-series wavelets. The vectors indicate the phase difference between the time-series. A vector pointing to the right side indicates an in-phase relationship and for positive correlation in the wavelet coherence plot whilst a vector to the left stands for anti-phase and for negative correlation in the wavelet coherence plot. The *x*-axis refers to time. The *y*-axis is the wavelet period in years. The thick black contour designates the 5% significance level against red noise. The cone of influence (COI) where edge effects might distort the results is shown as a lighter shade. (A) Cross-wavelet plot showing common spectrum power of ENSO and plague incidence anomalies time-series. Red denotes areas of high common power, blue of low common power. (B) Wavelet transform coherency plot of ENSO and plague incidence anomalies time-series showing periods of coherency between the signals. Red denotes areas of high coherency, blue of low coherency.

Cross-wavelet analysis (Figure 5A) indicates a high covariance between the power spectrums of ENSO and plague incidence anomalies throughout the time-series (red areas). The signals vary together with a 2–5 to 2–8 year cycle.

The wavelet coherence plot (Figure 5B) reveals that during parts of the time series the ENSO signal is strongly associated with plague incidence anomalies. However, the nature of this association varies in terms of time lag and direction. From the 1960s to the mid-1970s, there is a strong coherence between ENSO and plague incidence in the 2–5 year periodicity band. As in the cross-wavelet the vectors are pointing up, confirming plague leading ENSO by 9 months. In the 1980s, both signals show a significant coherence in the 3–5 year band. Left pointing vectors indicate that ENSO and plague incidence anomalies are in anti-phase (negatively correlated) over this period. In the final period of significant correlation, which lasts from the mid-1990s until almost the end, ENSO is strongly associated with plague incidence anomalies at a periodicity of about 1–2 years. The vectors denote that both time series are locked in phase (positively correlated) over this period.

#### Influence of the IOD on plague incidence anomalies

**Figure 6:**
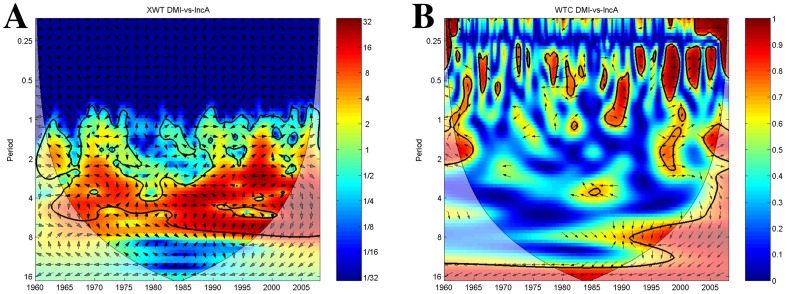
Plague incidence anomalies and IOD index time-series wavelets. The vectors indicate the phase difference between the time-series. A vector pointing to the right side indicates an in-phase relationship and for positive correlation in the wavelet coherence plot whilst a vector to the left stands for anti-phase and for negative correlation in the wavelet coherence plot. The *x*-axis refers to time. The *y*-axis is the wavelet period in years. The thick black contour designates the 5% significance level against red noise. The cone of influence (COI) where edge effects might distort the results is shown as a lighter shade. (A) Cross-wavelet plot showing common spectrum power of ENSO and plague incidence anomalies time-series. Red denotes areas of high common power, blue of low common power. (B) Wavelet transform coherency plot of ENSO and plague incidence anomalies time-series showing periods of coherency between the signals. Red denotes areas of high coherency, blue of low coherency.

Cross wavelet analysis (Figure 6A) shows high common power between plague incidence anomaly and the IOD in the 2–7 year period band throughout the time-series (red areas). However, the phase relationship between the signals varies greatly depending on the periodicity until the early 1980s when vectors point to the left at a 3–5 year band indicating the signals cycle in anti-phase. From 1995 this changes to an in-phase relationship between the IOD and plague incidence with a periodicity of 1.5 to 4.5 years.

The associated wavelet coherence plot (Figure 6B) confirms a short significant period of association around 1963/65 of negative correlation with a 1–2 year periodicity. Around the 7–12 year band another period of association is seen from the early 1990s to 2000 where the IOD is leading plague by 9 months (vectors pointing downward). In the late 1990s a positive association between anomalous plague incidence and the IOD emerges for a short time. This link cycles with a 1 to 2.5 year periodicity. Lastly, from 2003 to 2005, the IOD is lagging plague by around 3 months with a 1–2 year periodicity.

#### Influence of temperature and precipitation anomalies on plague incidence anomalies

**Figure 7:**
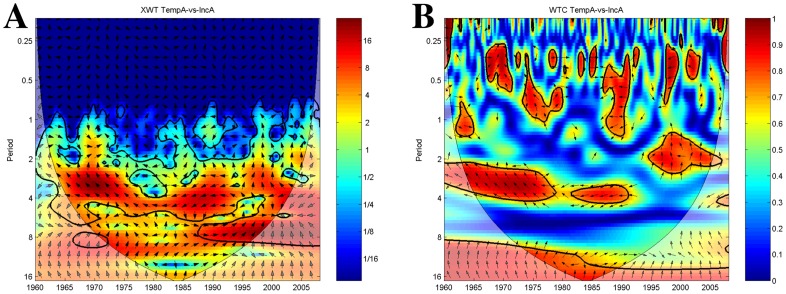
Temperature anomalies and plague incidence anomalies time-series wavelets. The vectors indicate the phase difference between the time-series. A vector pointing to the right side indicates an in-phase relationship and for positive correlation in the wavelet coherence plot whilst a vector to the left stands for anti-phase and for negative correlation in the wavelet coherence plot. The *x*-axis refers to time. The *y*-axis is the wavelet period in years. The thick black contour designates the 5% significance level against red noise. The cone of influence (COI) where edge effects might distort the results is shown as a lighter shade. (A) Cross-wavelet plot showing common spectrum power of ENSO and plague incidence anomalies time-series. Red denotes areas of high common power, blue of low common power. (B) Wavelet transform coherency plot of ENSO and plague incidence anomalies time-series showing periods of coherency between the signals. Red denotes areas of high coherency, blue of low coherency.

The cross-wavelet analyses show periods of common power of plague incidence anomalies with temperature anomalies throughout the time-series (Figures 7A). The signals cycle together with a 2–6 year periodicity and are anti-phase locked at the 4 year band extending throughout the time-series. Interestingly a second period of common power emerges at the end of the 1980 until the end of the time-series with a 7–10 year periodicity showing in-phase cycling of temperature and plague incidence at the edge of the COI. The coherency analysis (Figure 7B) shows a strong association between temperature and incidence anomalies from the 1960s to early 1990 with a 2–4 year period, followed by an association at a higher periodicity of 24–30 months from the mid 1990s until the end. Until the 1990s both signals appear to be almost in anti-phase indicating negative correlation. Vectors are pointing left and left-upward so the signals vary in anti-phase and with a 4–5 month lag respectively. After the 1990s a strong coherence around the 2 year band emerges with vectors denoting that the temperature anomaly series lags the plague incidence series by about 6 months.

**Figure 8:**
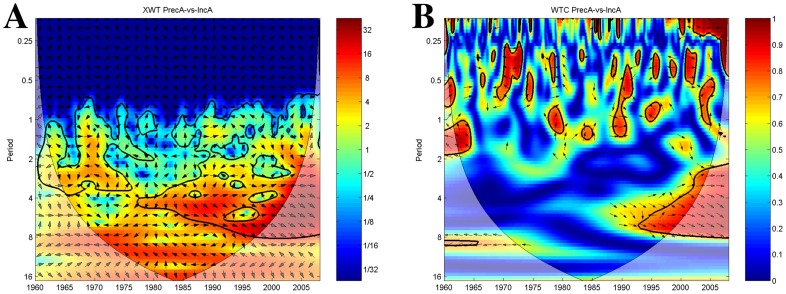
Precipitation anomalies and plague incidence anomalies time-series wavelets. The vectors indicate the phase difference between the time-series. A vector pointing to the right side indicates an in-phase relationship and for positive correlation in the wavelet coherence plot whilst a vector to the left stands for anti-phase and for negative correlation in the wavelet coherence plot. The *x*-axis refers to time. The *y*-axis is the wavelet period in years. The thick black contour designates the 5% significance level against red noise. The cone of influence (COI) where edge effects might distort the results is shown as a lighter shade. (A) Cross-wavelet plot showing common spectrum power of ENSO and plague incidence anomalies time-series. Red denotes areas of high common power, blue of low common power. (B) Wavelet transform coherency plot of ENSO and plague incidence anomalies time-series showing periods of coherency between the signals. Red denotes areas of high coherency, blue of low coherency.

For precipitation the cross-wavelet analysis reveals significant common power from the 1980s onwards with increasing bandwidth from 4 years to 2–8 years cycling mostly in-phase (Figure 8A). The associated wavelet coherence plot only shows short intermittent periods of coherency between signals until the mid 1990 when precipitation anomalies and plague incidence anomalies show significant positive association at the 7 year band extending to a 30 months–8 year band by the end of the time-series (Figure 8B).

## Discussion

It is widely accepted that ENSO and IOD are dominant drivers of earth's year-to-year climate variability, and have wide-ranging implications for public health, including influencing the periodicity of many infectious diseases [26], [27], [28], [29], [30], [31]. This study is the first, however, to demonstrate an influence of global climate drivers on plague incidence in Madagascar.

The results demonstrate a strong association between ENSO and plague in Madagascar, a country where about one third of the world's human cases occur and there is still significant mortality from the disease. For much of the time series ENSO cycles on a 2–5 year time scale (later 2–7 years). This appears to lead to a similar periodicity (also changing with time) in the occurrence of plague. However, the association is non-stationary: early in the time series, plague leads ENSO by a few months; later they are anti-phase; and at the end of the time series they are in phase. An analysis of the climate conditions around the time of high and low plague anomalies found evidence for larger plague outbreaks being associated with increasing ENSO signal (i.e. increasing El Niño conditions).

Our analyses find some association, also non-stationary, between IOD and plague. This association is less strong, however, than the association of ENSO and plague.

ENSO and IOD are unlikely to directly affect plague in Madagascar. Instead they influence local climate which in turn affects the disease. In this study we demonstrate that ENSO and IOD influence temperature in Madagascar and that temperature cycles are associated with plague cycles. Furthermore, the pattern of association between ENSO and temperature in terms of changes in lag and periodicity is similar to the pattern of association between the cycles of plague and temperature. We did not find a clear effect of ENSO or IOD on precipitation; and we found only limited evidence for association between cycles of precipitation and plague. However, while ENSO and IOD may have limited influence on precipitation, we nevertheless found that larger than usual plague outbreaks were significantly associated with rainier than usual years. Hence, the effects of ENSO and IOD on plague appear to be mediated more by temperature, but precipitation also undoubtedly influences plague in Madagascar.

During the late 1990s, ENSO events became progressively stronger, increasing in both frequency and amplitude. From 1976, a shift towards warmer and wetter conditions in the tropical Pacific was detected, with widespread climatic and ecological consequences [32]. Thus, changes in the association between ENSO, sea surface temperature and several diseases may be explained by their modulation by the decadal background and changes in variability. The analysis of ENSO and its association with plague incidence revealed a relationship between plague anomalies and an increase in the intensity of ENSO events and precipitation.

In our study the correlation between ENSO and plague turned from weakly positive to strongly negative and finally to positive again while the association between incidence and the IOD changed from negative to positive and became stronger with time. This non-stationarity is likely caused by changes to the lags in the response of Madagascar's climate to ENSO/IOD, or changes to the lags in the response of plague to local climatic conditions. The epidemiology of plague is sufficiently complex that the lag between climate and plague incidence may be long; as, for example, rodent and flea populations respond to favourable conditions. Thus, cycling of ENSO and plague in phase during the later part of the time-series does not necessarily imply that plague was responding immediately to ENSO. Instead, it could be that its response is an entire cycle behind (1–2 years). Equally, at first sight it seems hard to reconcile that ENSO affects plague, and yet in the early part of the time series plague leads (comes before) ENSO. It is possible, however, that plague is actually most of a cycle behind ENSO.

The plague system is highly seasonal in the plague focus area which suggests that the dry and cold months from May to September are not favourable for transmission. However, there is a changing relationship between ENSO and its effects on temperature and in turn on plague. Until the 1980s ENSO affects temperature positively on the island 12 to 18 months later, while temperature is associated negatively with plague. From then onwards until the end of the time-series, the temperature response to ENSO accelerates to 8–9 months. This shift in response is most likely related to changes in the frequency and magnitude of ENSO events and a warmer decadal background as shown by the overall increase in temperature.

At times ENSO and IOD may interact in their effects on plague. During the first phase of positive association between ENSO and incidence from 1960 to 1975, a period dominated by La Niña events, notable plague outbreaks are related to El Niño causing warmer than usual temperatures during a time of cooler and drier conditions usually brought by La Niña. In the second phase during the 1980s, plague is negatively associated with ENSO during a time when the temperature response to ENSO accelerates, changing the timing of an increase in temperature. Finally, from 1995 onwards, the intensity and magnitude of ENSO events increases drastically and plague shows positive correlation with ENSO again. At the same time the IOD starts impacting on plague probably via its effects on temperature 6 months later and almost immediate effects on rainfall. Together, positive ENSO and IOD events are creating warmer and wetter conditions. This is exemplified by 1997 which saw one of the largest positive plague incidence anomalies, and was the year with the strongest El Niño and positive IOD in the time-series.

The intensity of ENSO and IOD events and corresponding increases in temperature, as well as increases in rainfall, show strong associations with an increase in plague incidence in Madagascar. The seasonality of plague confirms the strong link between disease transmission and optimal environmental conditions, via their effects on vector and host. Higher temperature and increased precipitation during the cold and dry season in Madagascar are likely to increase flea survival and shorten flea development time [33], [34], [35], [36]. An ENSO event influencing the temperature around this time of year would therefore create more favourable conditions for plague transmission.

Changes in the climate–disease relationship over time, as detected here, have been found for certain other diseases like cholera [37], most likely modulated by long-term climate change effects and their influence on epidemiological systems.

Plague incidence has also been linked to global climate in other parts of the world, and several studies have hypothesized about the potential mechanisms. Temperature thresholds exist for pathogen survival and transmission, and temperature and precipitation affect the environment of both vector and rodent host. In China, an increased rate of human plague at the province level was associated with the Southern Oscillation Index and Sea Surface Temperature of the tropic Pacific east of the equator [38]. Similarly, inter-annual variability of disease incidence in the Americas correlates with global climate phenomena, with the link hypothesised to be caused by effects on the rodent host community [39], [40], [41]. In the US the association between global climate and plague was shown to depend on time-lagged precipitation events, presumably increasing rodent populations via food availability, and on relatively cool summer temperatures during the plague transmission season, potentially increasing the abundance of infectious fleas [21]. Therefore a coherency of ENSO and plague incidence is most likely due to the influence of ENSO on temperature which in turn affects host and vector ecology and transmission potential [2], [41], [42].

There are of course other drivers for plague, which have not been considered here. These might also be related to climate effects and act on plague indirectly through anthropogenic and socioeconomic factors such as poverty, migration and cultural practises, all of which can influence disease transmission risks [43], [44], [45]. Global climate influences an array of factors which affect the epidemiology of plague, many of which will depend on both ecological and anthropogenic characteristics. The implications of a link between human plague and ENSO and IOD in Madagascar are exceedingly complex but this study leads the way to understanding the relationship between large scale climate and plague in a country where one third of the world's cases occur.

## Supporting Information

Figure S1 **Monthly human plague incidence time-series for Madagascar for the period 1960–2008.** Incidence was computed using 5-yearly human population growth estimates from the United Nations and the last population census from 1993 and the number of plague cases per month. Incidence is presented per 100000.
(EPS)

Figure S2 **Monthly anomaly time-series of DMI (IOD index) for the period 1960–2008.** Positive values are depicted in red, negative values are blue.
(TIF)

Figure S3 **Monthly anomaly time-series of temperature (NCEP) for the period 1960–2008.** Positive values are depicted in red, negative values are blue.
(TIF)

Figure S4 **Monthly anomaly time-series of precipitation (NCEP) for the period 1960–2008.** Positive values are depicted in red, negative values are blue.
(TIF)

Figure S5 **1D plot of the monthly DMI index (black) and the monthly filtered plague incidence anomalies (red) for the period 1960–2008.**
(TIF)

Figure S6 **1D plot of the monthly temperature anomalies (red) and the monthly filtered plague incidence anomalies (black) for the period 1960–2008.**
(TIF)

Figure S7 **1D plot of the monthly precipitation anomalies (blue) and the monthly filtered plague incidence anomalies (black) for the period 1960–2008.**
(TIF)

Figure S8 **1D plots of the monthly JMA (ENSO index) (black) and (A) the monthly precipitation anomalies (blue) for the period 1960–2008 (B) the monthly temperature anomalies (red) for the period 1960–2008.**
(TIF)

Figure S9 **1D plots of the monthly DMI (IOD index) (black) and (A) the monthly precipitation anomalies (blue) for the period 1960–2008 (B) the monthly temperature anomalies (red) for the period 1960–2008.**
(TIF)

Figure S10 **ENSO (JMA index), time-series decomposition.** Continuous wavelet decomposition plot of the JMA index time-series. The *x*-axis refers to time. The *y*-axis is the wavelet period in years. The thick black contour designates the 5% significance level against red noise. The cone of influence (COI) where edge effects might distort the results is shown as a lighter shade. Red denotes areas of high power, blue of low power.
(TIF)

Figure S11 **IOD (DMI index), time-series decomposition.** Continuous wavelet decomposition plot of the DMI index showing IOD time-series. The *x*-axis refers to time. The *y*-axis is the wavelet period in years. The thick black contour designates the 5% significance level against red noise. The cone of influence (COI) where edge effects might distort the results is shown as a lighter shade. Red denotes areas of high power, blue of low power.
(TIF)

Figure S12 **Temperature time-series decomposition.** Continuous wavelet decomposition plot showing temperature time-series. The *x*-axis refers to time. The *y*-axis is the wavelet period in years. The thick black contour designates the 5% significance level against red noise. The cone of influence (COI) where edge effects might distort the results is shown as a lighter shade. Red denotes areas of high power, blue of low power.
(TIF)

Figure S13 **Precipitation time-series decomposition.** Continuous wavelet decomposition plot showing precipitation time-series. The *x*-axis refers to time. The *y*-axis is the wavelet period in years. The thick black contour designates the 5% significance level against red noise. The cone of influence (COI) where edge effects might distort the results is shown as a lighter shade. Red denotes areas of high power, blue of low power.
(TIF)
