# Supplementary figures and images for: A Non-Stationary Relationship between Global Climate Phenomena and Human Plague Incidence in Madagascar
Source: PLoS Negl Trop Dis. 2014 Oct 9;8(10):e3155. doi: 10.1371/journal.pntd.0003155 (PMC4191945; doi:10.1371/journal.pntd.0003155)

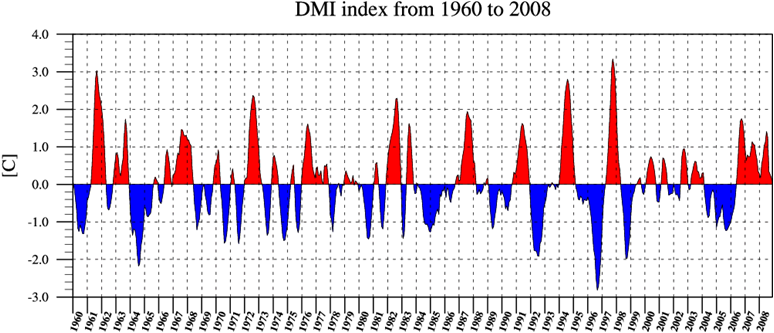

Supplement: Figure S2 — Monthly anomaly time-series of DMI (IOD index) for the period 1960–2008. Positive values are depicted in red, negative values are blue. (TIF) [file pntd.0003155.s002.tif]

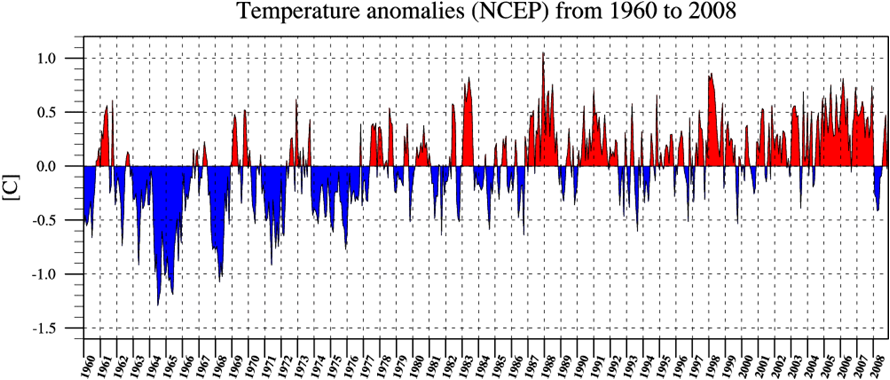

Supplement: Figure S3 — Monthly anomaly time-series of temperature (NCEP) for the period 1960–2008. Positive values are depicted in red, negative values are blue. (TIF) [file pntd.0003155.s003.tif]

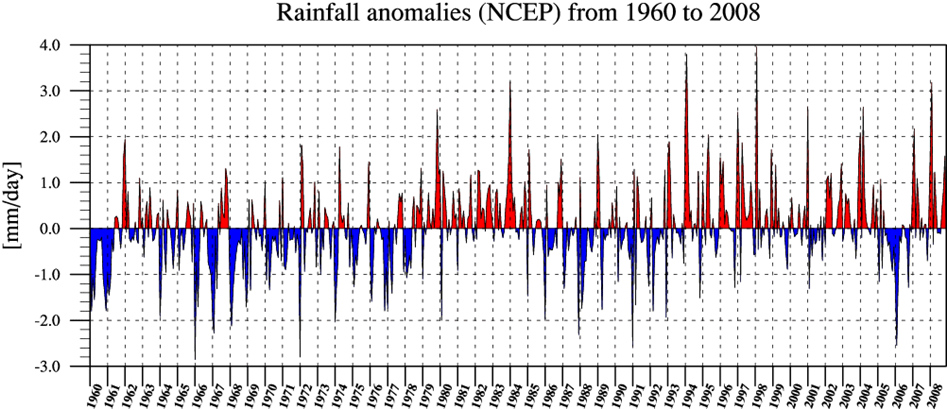

Supplement: Figure S4 — Monthly anomaly time-series of precipitation (NCEP) for the period 1960–2008. Positive values are depicted in red, negative values are blue. (TIF) [file pntd.0003155.s004.tif]

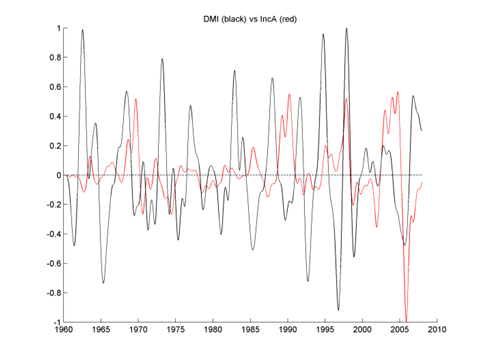

Supplement: Figure S5 — 1D plot of the monthly DMI index (black) and the monthly filtered plague incidence anomalies (red) for the period 1960–2008. (TIF) [file pntd.0003155.s005.tif]

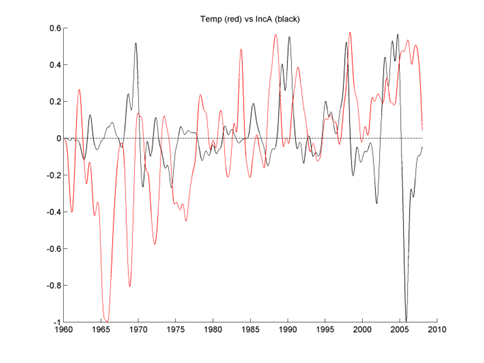

Supplement: Figure S6 — 1D plot of the monthly temperature anomalies (red) and the monthly filtered plague incidence anomalies (black) for the period 1960–2008. (TIF) [file pntd.0003155.s006.tif]

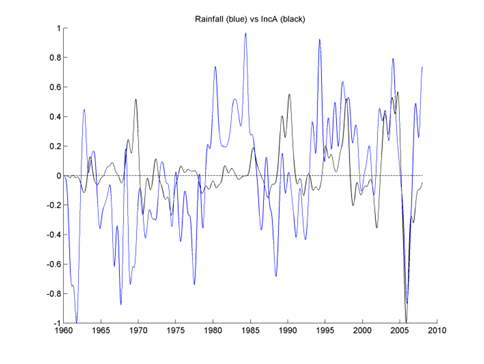

Supplement: Figure S7 — 1D plot of the monthly precipitation anomalies (blue) and the monthly filtered plague incidence anomalies (black) for the period 1960–2008. (TIF) [file pntd.0003155.s007.tif]

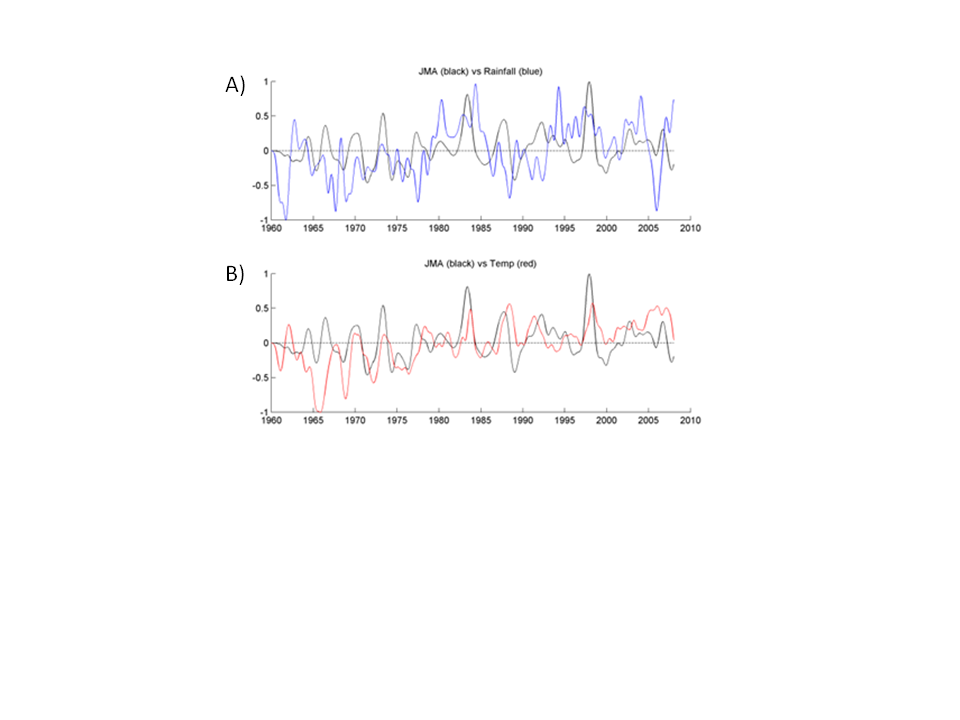

Supplement: Figure S8 — 1D plots of the monthly JMA (ENSO index) (black) and (A) the monthly precipitation anomalies (blue) for the period 1960–2008 (B) the monthly temperature anomalies (red) for the period 1960–2008. (TIF) [file pntd.0003155.s008.tif]

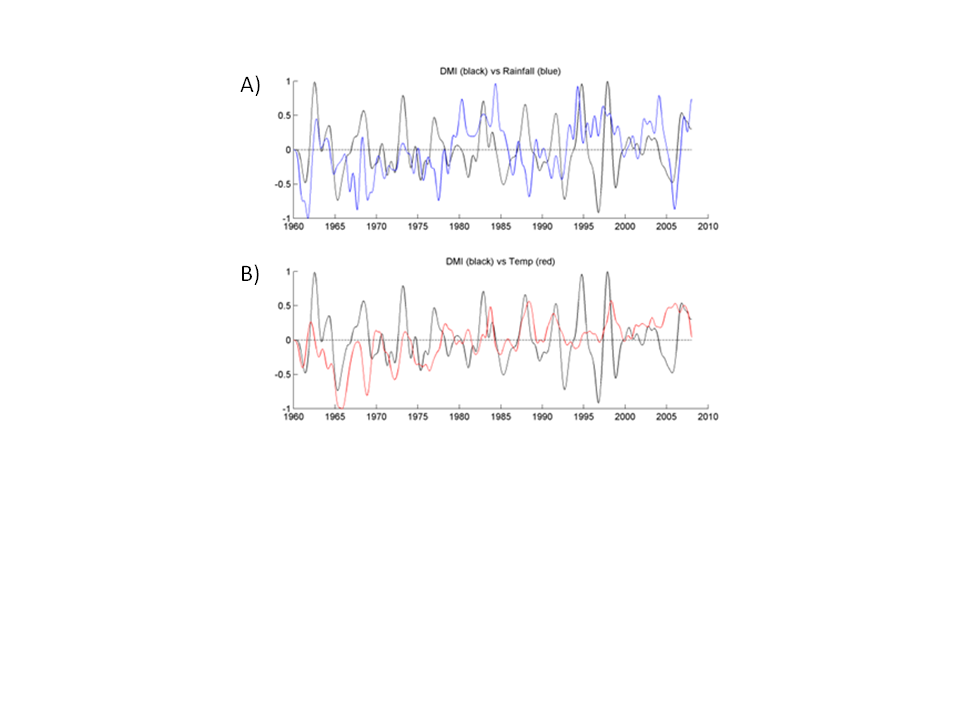

Supplement: Figure S9 — 1D plots of the monthly DMI (IOD index) (black) and (A) the monthly precipitation anomalies (blue) for the period 1960–2008 (B) the monthly temperature anomalies (red) for the period 1960–2008. (TIF) [file pntd.0003155.s009.tif]

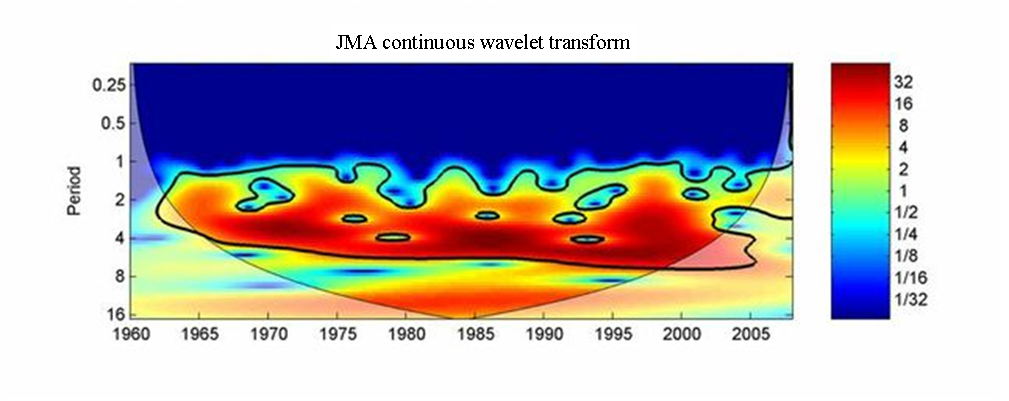

Supplement: Figure S10 — ENSO (JMA index), time-series decomposition. Continuous wavelet decomposition plot of the JMA index time-series. The x-axis refers to time. The y-axis is the wavelet period in years. The thick black contour designates the 5% significance level against red noise. The cone of influence (COI) where edge effects might distort the results is shown as a lighter shade. Red denotes areas of high power, blue of low power. (TIF) [file pntd.0003155.s010.tif]

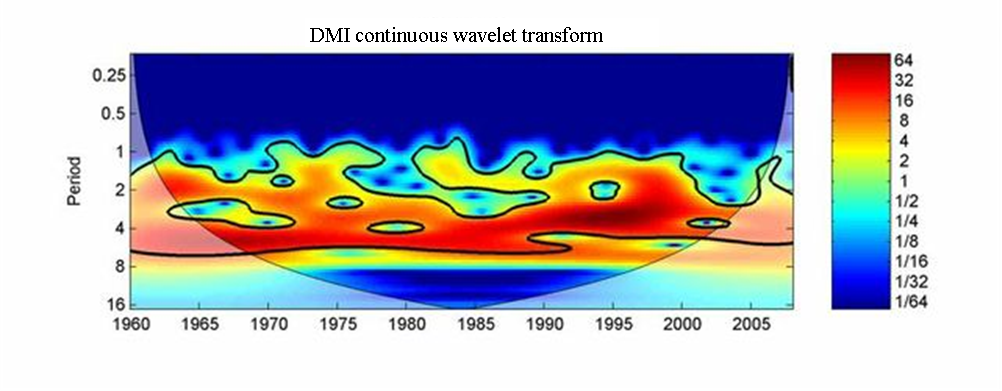

Supplement: Figure S11 — IOD (DMI index), time-series decomposition. Continuous wavelet decomposition plot of the DMI index showing IOD time-series. The x-axis refers to time. The y-axis is the wavelet period in years. The thick black contour designates the 5% significance level against red noise. The cone of influence (COI) where edge effects might distort the results is shown as a lighter shade. Red denotes areas of high power, blue of low power. (TIF) [file pntd.0003155.s011.tif]

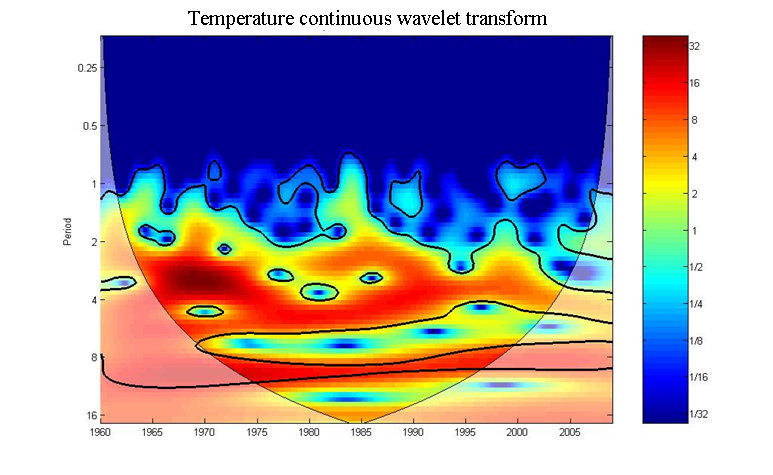

Supplement: Figure S12 — Temperature time-series decomposition. Continuous wavelet decomposition plot showing temperature time-series. The x-axis refers to time. The y-axis is the wavelet period in years. The thick black contour designates the 5% significance level against red noise. The cone of influence (COI) where edge effects might distort the results is shown as a lighter shade. Red denotes areas of high power, blue of low power. (TIF) [file pntd.0003155.s012.tif]

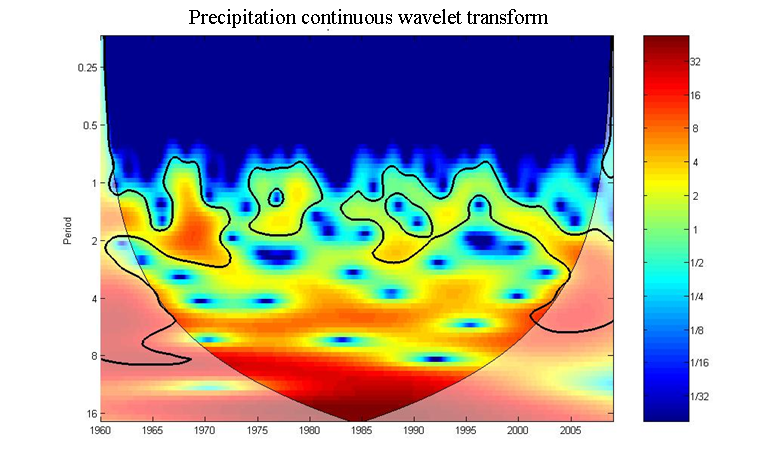

Supplement: Figure S13 — Precipitation time-series decomposition. Continuous wavelet decomposition plot showing precipitation time-series. The x-axis refers to time. The y-axis is the wavelet period in years. The thick black contour designates the 5% significance level against red noise. The cone of influence (COI) where edge effects might distort the results is shown as a lighter shade. Red denotes areas of high power, blue of low power. (TIF) [file pntd.0003155.s013.tif]
